# Phase-based metamorphosis of diffusion lesion in relation to perfusion values in acute ischemic stroke

**DOI:** 10.1016/j.nicl.2015.07.007

**Published:** 2015-08-01

**Authors:** Islem Rekik, Stéphanie Allassonnière, Marie Luby, Trevor K. Carpenter, Joanna M. Wardlaw

**Affiliations:** a Division of Neuroimaging Sciences, Brain Research Imaging Centre, University of Edinburgh, UK; b Brain Research Imaging Centre, SINAPSE Collaboration, UK; c CMAP, Ecole Polytechnique, Route de Saclay, 91128 Palaiseau, France; d National Institute of Neurological Disorders and Stroke, National Institutes of Health, Bethesda, MD, USA; e Seton/UT Southwestern Clinical Research Institute of Austin, Department of Neurology and Neurotherapeutics, UT Southwestern Medical Center, Austin, TX, USA

**Keywords:** Metamorphosis, Ischemic stroke, Lesion evolution, Diffusion imaging, Perfusion imaging, Magnetic resonance imaging

## Abstract

Examining the dynamics of stroke ischemia is limited by the standard use of 2D-volume or voxel-based analysis techniques. Recently developed spatiotemporal models such as the 4D metamorphosis model showed promise for capturing ischemia dynamics. We used a 4D metamorphosis model to evaluate acute ischemic stroke lesion morphology from the acute diffusion-weighted imaging (DWI) to final T2-weighted imaging (T2-w). In 20 representative patients, we metamorphosed the acute lesion to subacute lesion to final infarct. From the DWI lesion deformation maps we identified dynamic lesion areas and examined their association with perfusion values inside and around the lesion edges, blinded to reperfusion status. We then tested the model in ten independent patients from the STroke Imaging Repository (STIR). Perfusion values varied widely between and within patients, and were similar in contracting and expanding DWI areas in many patients in both datasets. In 25% of patients, the perfusion values were higher in DWI-contracting than DWI-expanding areas. A similar wide range of perfusion values and ongoing expansion and contraction of the DWI lesion were seen subacutely. There was more DWI contraction and less expansion in patients who received thrombolysis, although with widely ranging perfusion values that did not differ. 4D metamorphosis modeling shows promise as a method to improve use of multimodal imaging to understand the evolution of acute ischemic tissue towards its fate.

## Highlights

• Modeling ischemic stroke lesion metamorphosis using MR diffusion/perfusion imaging
• Using spatiotemporal metamorphosis model for simulation
• Examining the native diffusion lesion core growth in relation to perfusion values
• Heterogeneity of perfusion values and a non-specific influence on lesion dynamics

## Graphical abstract

**Figure:**
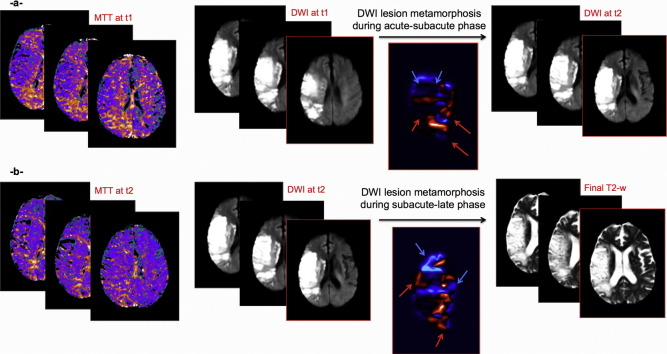

## 1 Introduction

The change in ischemic stroke lesions from acute presentation to final tissue damage is highly variable between individual patients as seen on magnetic resonance diffusion and perfusion imaging. Following the occlusion of a cerebral artery, ischemic tissue damage is seen as hyperintense on diffusion-weighted imaging (DWI) often within a larger area of hypoperfused at-risk, but potentially reversible tissue ischemia, detectable on perfusion-weighted imaging (PWI). Thereafter the ischemic tissue may grow or diminish depending on known and unknown factors. Subsequent growth of the lesion core, considered to be represented by DWI, is generally attributed to persistently reduced perfusion values around the core, whereas recovery of ischemic tissue is generally attributed to improvement in perfusion (Wardlaw, 2010).

Many imaging studies have investigated stroke lesion evolution mainly using 2D lesion volume or voxel-based analyses, but these may not capture the full spatiotemporal dynamics of perfusion and diffusion lesions as they may under-sample information about the location, direction or magnitude of the lesion dynamics in space and time (Rekik et al., 2012). Recently, we applied 4D shape deformation modeling methods to examine the highly contracting and expanding areas in DWI and PWI lesions (Rekik et al., 2013, 2014).

Of theses methods, the metamorphosis model (Trouvé and Younes, 2005; Younes, 2010; Rekik et al., 2014) handled both multi-component and solitary lesions and incorporated image intensity values from different sequences, and demonstrated elegance and accuracy of deforming the source image into a subsequent image, while tracking, point by point, a) the image intensity values inside and outside the lesion edges and b) the velocity of lesion deformation between timepoints. Notably, the proposed metamorphosis model in (Rekik et al., 2014) could follow ischemic stroke lesion change in perfusion weighted imaging from the acute to final infarct. It enabled to explore the perfusion dynamics in ischemic stroke and their relation to final T2-w lesion outcome (at ≥ 1 month). However, the role of diffusion weighted imaging, which is fundamental to understanding stroke dynamics, was overlooked. In this paper, we aim to investigate diffusion lesion local dynamic changes in relation to perfusion values in the affected hemisphere.

By applying this model to longitudinal images, the present study aims to: (1) model changes in the acute ischemic DWI lesion from the acute timepoint into the final infarct lesion, in both solitary and multi-component lesions; and (2) extract measurements of the most dynamic parts of the lesion to see the most rapid or largest areas of the DWI lesion expansion/contraction areas in relation to PWI values and clinical features such as stroke severity. We tested our model on stroke imaging data acquired in an observational study in one center (Rivers et al., 2006; Kane et al., 2007) and then validated the model in multicenter data obtained from STIR (Ali et al., 2007).

## 2 Material and methods

### 2.1 Patient selection

#### 2.1.1 Development dataset

We first applied the metamorphosis model to 20 representative patients from a prospective observational study of MRI in hyperacute stroke (Rivers et al., 2006; Kane et al., 2007). Patients were first imaged < 6 h of stroke and represented a typical range of stroke severities (NIHSS, median = 10, IQR: 6–14), ages (74.9 ± 9.2 years), acute DWI lesion volumes (34.6 ± 32.2 cm^3^) and mean transit time (MTT) volumes (126.6 ± 102.2 cm^3^). None of the 20 patients received rt-PA treatment, thus they represent the natural history of stroke lesion evolution, including any effects of spontaneous reperfusion. We included patients who had DWI images at acute (~ 5 h) and subacute (~ 5 ± 1 days) timepoints after stroke, a perfusion mean transit time (MTT) map at least at the acute timepoint, and T2-w lesion at ≥ 1 month after stroke. All patients had an MTT lesion at the first timepoint but only 12 had an MTT lesion visible at the second timepoint. Twelve patients had multi-component DWI/MTT lesions and eight had solitary lesions.

#### 2.1.2 Exploratory dataset

We selected from STIR the first 10 of 290 potential cases with three MRI scans at acute (< 6 h), subacute (5 days) and final (≥ 1 month). The first 10 patients that met the study criteria (age 59.6 ± 16.4 years, median admission NIHSS of 7 (IQR: 5–12)) had all received standard IV tPA thrombolysis. All had perfusion imaging < 6 h but perfusion imaging was included per protocol at subacute (5 days).

### 2.2 MRI pre-processing

We used the MTT perfusion map as it is easily obtained and generally shows the PWI lesion as large (Rivers et al., 2006; Kane et al., 2007). The modeling was blind to all clinical data and imaging values. Arterial recanalization status and collaterals were not taken into account in the modeling as angiographic data were not available for all patients. STIR exploratory data were processed identically to the derivation data unless stated otherwise. Full details of image acquisition and processing were described previously (Rivers et al., 2006; Luby et al., 2006). We obtained MTT areas from the contralateral hemisphere by mirror reflection of the MTT lesion to the unaffected hemisphere. For each patient, we generated relative MTT (rMTT) lesion maps by dividing the value of each lesion voxel by the mean perfusion value of the contralateral MTT values. The resulting intensity ‘rMTT’ has no unit. An expert radiologist visually checked that tissue swelling did not distort the DWI lesion boundary.

### 2.3 Two-image based metamorphosis model

In our previous work (Rekik et al., 2014), we extended the image-to-image metamorphosis into a spatiotemporal metamorphosis that exactly fits the baseline image to subsequent observations in an ordered set ℑ = {*I*_0_, *I*_1_, …, *I*_*T*_} of images, which we applied to perfusion data in acute stroke. This model registers one source image to a target image while estimating two optimal evolution paths linking these images: (1) a geometric path encoding the smooth velocity of the deformation of one image into another, and (2) a photometric path representing the variation in image intensity. Both paths characterize the dynamics of the image metamorphosis from the source to the target image in small discrete time and space intervals.

Basically, a baseline image *I*_0_ morphs under the action of a velocity vector field *v_t_* that advects the scalar intensity field *I_t_* (i.e. time-evolving image intensity) (Trouvé and Younes, 2005). Solving the advection equation with a residual allows to estimate both image intensity evolution and the velocity at which it moves.

We estimated the optimal metamorphosis path (*I_t_*, *I_v_* starting at *I*_0_, while constraining it to smoothly and exactly go through any available intermediate observation, till reaching the final observation *I_T_*. This was achieved through minimizing the following cost functional *U* using a standard alternating steepest gradient descent algorithm (Rekik et al., 2014):

$$U\left(I,v\right)=\int_{0}^{T}|v\_t{|}_{V}^{2}dt+\frac{1}{\sigma^{2}}\int_{0}^{T}{\left|\frac{dI\left(t\right)}{dt}+\nabla I_{t}.v_{t}\right|}_{L^{2}}^{2}dt$$

*σ* weighs the trade-off between the deformation smoothness (first term) and fidelity-to-data (second term). The term ∇*I*_*t*_. *v*_*t*_ represents the spatial variation of the moving image *I_t_* in the direction *v_t_*. Furthermore, the moving intensity scalar field *I_t_* is defined under the action of the diffeomorphism (invertible smooth mapping) *ϕ_t_* on a baseline image *I*_0_ : *I*_*t*_ = *ϕ*_*t*_. *I*_0_. We associated to the action *ϕ* a velocity *v* that satisfies the flow equation rooted in the in-vogue large deformation diffeomorphic metric (LDDMM) framework (Trouvé, 1998):

$$\left\{\begin{array}{l} \frac{d_{\phi t}}{dt}=v\left(\phi_{t}\left(x\right)\right),\;t\in \left[0;T\right]\\ \phi_{0}\left(x\right)=x \end{array}\right)\text{.}$$

In the present study, we used the estimated velocity vector field *v_t_* to estimate the total DWI lesion deformation map in two phases.

1) In the first phase, we morphed acute (< 6 h) DWI lesion to subacute (~ 5 days) DWI lesion in 20 patients; and
2) In the second phase, we morphed the subacute (~ 5 days) DWI lesion into the final T2-w (≥ 1 month) in the 12/20 patients with subacute perfusion imaging. Retaining these two distinct phases, ‘acute to subacute’ and ‘subacute to final’, facilitated testing of acute separately from subacute clinical information against the lesion parameters.

### 2.4 Extracting highly dynamic regions of DWI lesion

**Fig. 1:**
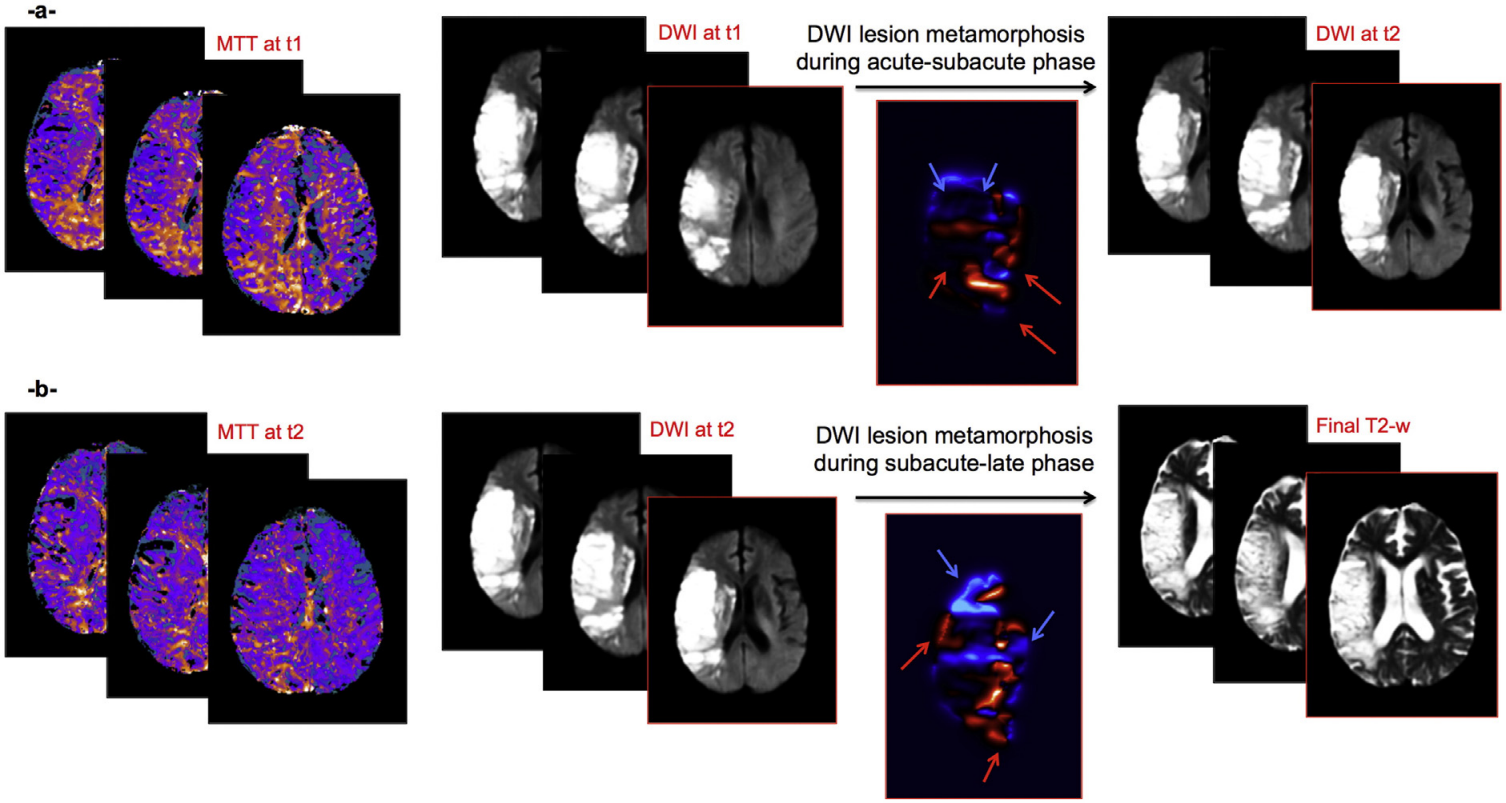
Phase-based DWI lesion metamorphosis in light of spatiotemporal perfusion values. (a) Acute–subacute phase data: Axial images of acute MTT (left), acute DWI (middle) and subacute DWI (right). (b) Subacute-final phase data: Axial images of subacute MTT image (left), subacute DWI image (middle) and final T2-w image (right). During acute–subacute phase, we metamorphose acute DWI lesion to subacute DWI lesion. During subacute-final phase, we deform the latter into final T2-w lesion. We estimate the deformation maps for both phases of DWI lesion evolution (images under the black arrows). The red arrows point to highly expanding (red) areas and blue arrows point to highly contracting (blue) areas.

For both phases, in each patient, we generated a total 3D lesion deformation map, computed as the squared sum of the estimated speed along the metamorphosis path, and identified contracting and expanding DWI regions (as the ‘negative’ and ‘positive’ deformation values respectively) during each phase (Fig. 1). In the exploratory dataset (STIR), we were only able to estimate the acute to subacute phases since subacute perfusion imaging was not available for all 10 patients. We then automatically thresholded the two total metamorphosis deformation maps generated for the acute-subacute and subacute-late phases of DWI lesion evolution to compute the proportion by volume of the total DWI lesion boundary that was rapidly contracting or expanding for the acute-subacute and subacute-late phases.

### 2.5 rMTT values relation to DWI lesion dynamics

**Fig. 2:**
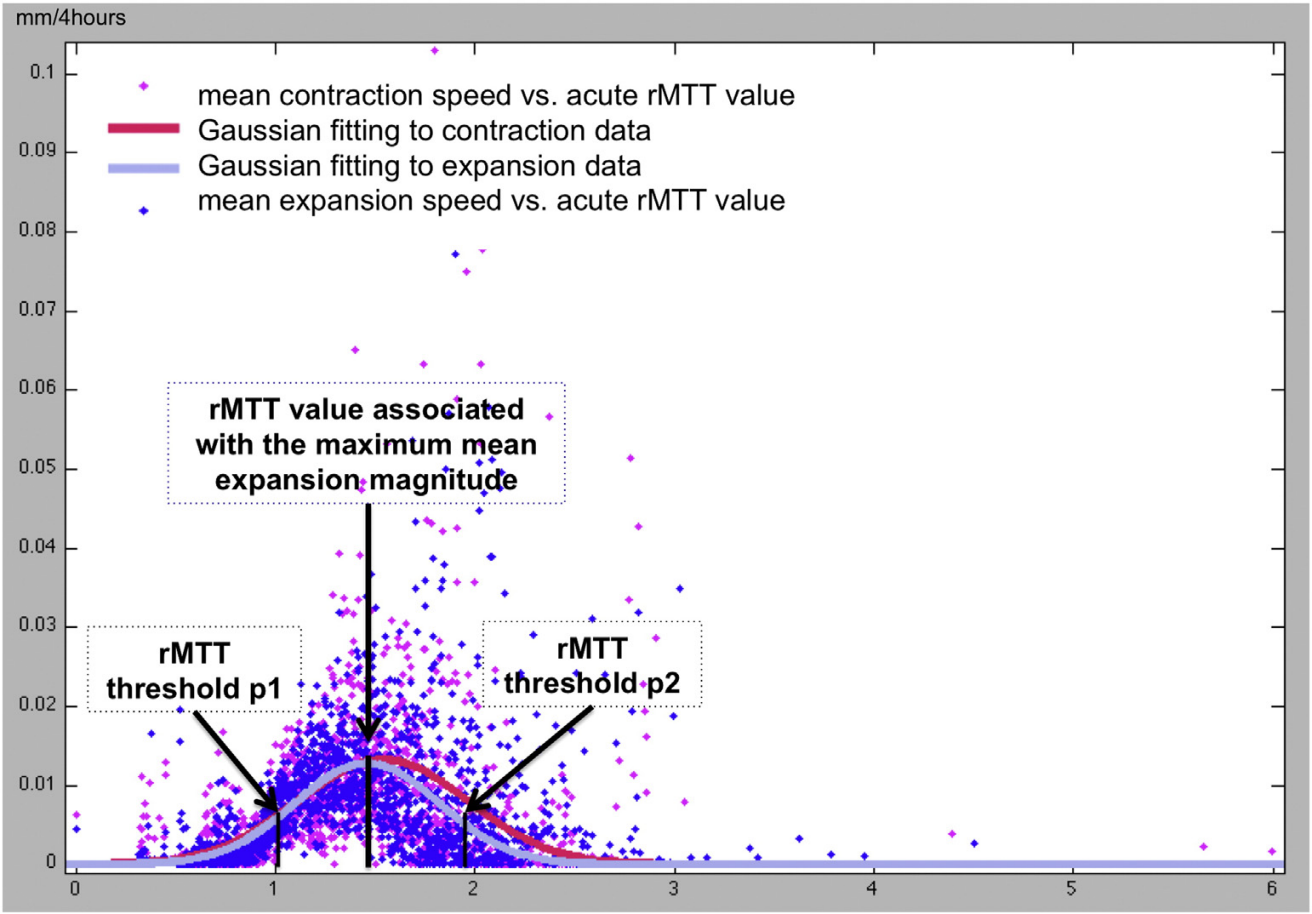
Distribution of rMTT perfusion values (x-axis, ratio, therefore no unit) and the DWI lesion mean deformation magnitude (y-axis mm/4 h) between the acute and subacute images for one patient. Blue dots are rMTT values in expanding areas of the DWI lesion and pink dots are rMTT values in contracting areas of the DWI lesion. The peak of the fitted Gaussian curve (purple line) represents the rMTT value associated with the maximum mean contraction magnitude. The black arrows point to two perfusion thresholds on the purple curve: p1 representing the peak of the Gaussian curve minus its standard deviation and p2 the Gaussian peak plus its standard deviation. The perfusion confidence interval from p1 to p2 defines a range for perfusion values associated with the most rapidly contracting diffusion areas. Same parameters are estimated from the red line fitting the pink dot distribution (for expansion).

For each patient, for both phases, for every rMTT voxel value within the acute perfusion image we computed the mean amount of DWI lesion contraction or expansion. We then plotted the acute rMTT values against their corresponding mean DWI contraction or expansion magnitudes (Fig. 2). In most cases, both resulting rMTT distributions were Gaussian. Therefore, we used Gaussian least squares fit to approximate the relation between acute rMTT values and the mean amounts of DWI lesion deformation: one for contraction (purple curve in Fig. 2) and one for expansion (pink curve in Fig. 2). For phase one, the Gaussian fitting root-mean-square deviation (RMSE) reached 0.0035 ± 0.0042 for contraction and 0.006 ± 0.014 for expansion, noting that when the fitting is exact RMSE = 0 (no residuals or perfect test). For phase two, the data also best fitted a Gaussian distribution (RMSE = 0.0029 ± 0.0032 for contraction and 0.0039 ± 0.0058 for expansion). These Gaussian curves allowed us to estimate the rMTT values associated with rapidly expanding or contracting DWI regions—along with a confidence interval around these peak values: the upper bound represents the mean of the Gaussian curve minus its standard deviation and the lower bound represents the mean of the Gaussian curve plus its standard deviation.

## 3 Results

### 3.1 Derivation dataset lesion metamorphosis and perfusion values: acute to subacute phases

**Fig. 3:**
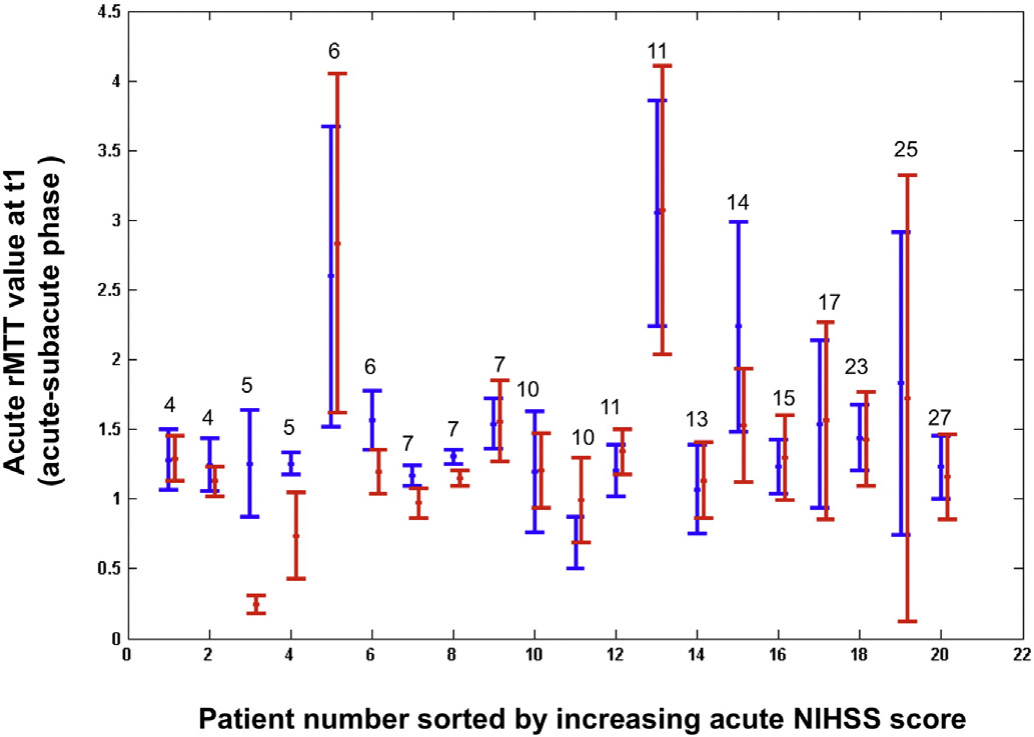
Acute rMTT values associated with rapidly deforming DWI lesion areas graphed for all patients—ordered left to right by increasing admission NIHSS score (values on top of the vertical bars). The center dot = rMTT values associated with the maximum of DWI lesion mean deformation magnitude (= peak of Gaussian curve in Fig. 2). The limits of the vertical blue and red bars represent the lower and the upper acute rMTT values (interval [p1, p2] in Fig. 2) associated with rapidly contracting (blue) vs. expanding (red) DWI lesion areas between the acute and subacute timepoints.

The model showed that the mean rMTT values in areas of DWI expansion across patients (mean 0.8 ± 0.82SD, maximum 3.18) were similar to mean rMTT values in areas of DWI contraction (mean 0.74 ± 0.63SD, maximum 2.17), Table 1. In general, the range of rMTT values in all parts of the DWI lesion boundary was wide such that in most of the 20 derivation dataset patients (15/20), the rMTT values in DWI lesion areas that were contracting were nearly identical to the values in DWI areas that were expanding (correlation coefficient r = 0.86, p = 0.8), shown as the overlap of the red and blue vertical bars in Fig. 3. Only in 5/20 patients (25%) were the blue and red vertical bars distinct, indicating that acute perfusion was clearly better in areas of DWI contraction than in areas of DWI expansion (Fig. 3). In some patients (7/20 in Fig. 3), the red bars extended beyond the blue bars indicating that perfusion values associated with most rapidly expanding DWI areas encompassed a wider range of MTT perfusion values than in rapidly contracting DWI areas.

### 3.2 Lesion metamorphosis and perfusion values: subacute to final phase

**Fig. 4:**
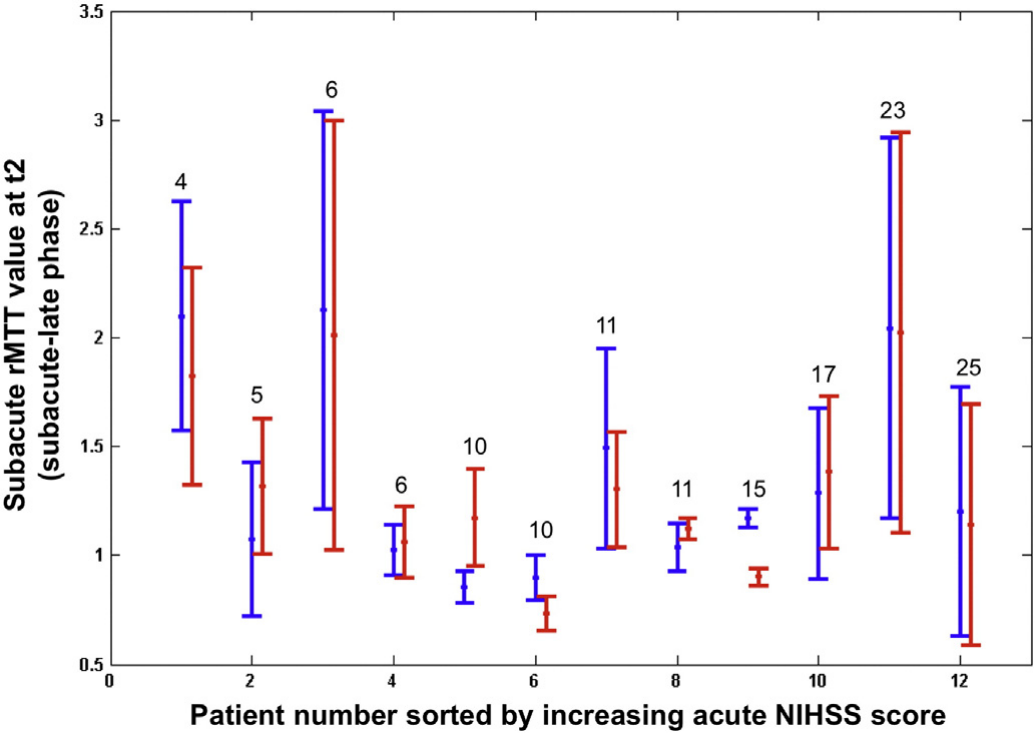
Subacute rMTT values associated with rapidly deforming DWI lesion areas graphed for all patients ordered left to right by increasing admission NIHSS score (values on top of the vertical bars). The center dot = rMTT value associated with the maximum of DWI lesion mean deformation magnitude. The limits of the vertical blue and red bars represent the confidence interval (in Fig. 2) associated with rapidly contracting (blue) vs. expanding (red) DWI lesion areas between the subacute and final timepoints.

A similar general pattern of rMTT values was seen in the 12 patients who had rMTT data available from the subacute to final phases (~ 5 days to > 1 month, Table 1, Fig. 4). The rMTT values were very similar in DWI expanding and contracting areas in most patients (r = 0.91, p = 0.8). In 3/12 patients (25%), subacute rMTT values in DWI contracting areas were higher than in DWI lesion expansion areas indicating better perfusion in contracting areas. Table 1 also indicates that a) DWI lesions were still expanding into some areas and regressing in others and b) perfusion values remain very variable during the subacute to final phase.

### 3.3 DWI dynamic evolution features

We assessed the proportion of the DWI lesion that highly contracted or expanded at each phase (Table 1). During both phases, 11/20 (55%) patients had more highly expanding than contracting areas, although the difference in median volumetric proportions of the DWI lesion was not significant (p = 0.62 for acute–subacute and p = 0.13 for subacute-final phases). Also some DWI lesions continued to expand rapidly in some areas and to contract in others in quite similar proportions, highlighting the dynamism of acute and subacute stroke lesions (Table 1).

### 3.4 DWI metamorphosis, perfusion and clinical features

During the acute–subacute phase, there was no association between acute NIHSS and rMTT values found in expanding (r = 0.11, p = 0.63) or contracting (r = 0.06, p = 0.77) DWI lesion areas. Similarly, during subacute-final phase, there was no association between admission NIHSS and rMTT values found in contracting (r = 0.007, p = 0.99) or expanding (r = − 0.021, p = 0.95) DWI lesion areas. We also investigated the association between the proportion of the DWI lesion volume that was highly contracting or expanding during acute–subacute and subacute-final phases and various clinical factors (acute NIHSS, acute MTT volume, acute DWI volume), but found no significant correlations. For all, we obtained: (i) acute–subacute phase: contraction r = 0.082, p = 0.731; expansion r = 0.258, p = 0.271 and (ii) subacute-final phase: contraction r = 0.181, p = 0.444; expansion r = 0.255, p = 0.277.

### 3.5 Evaluation of the model in the exploratory dataset from STIR

**Fig. 5:**
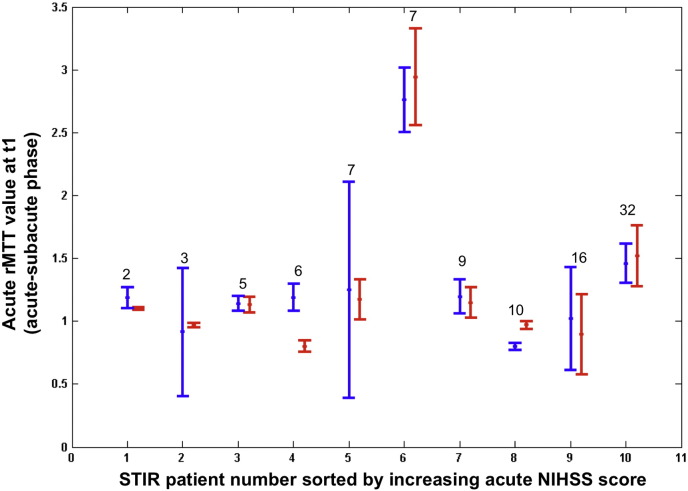
Acute rMTT values associated with rapidly deforming DWI lesion areas graphed for STIR patients ordered left to right by increasing admission NIHSS score (values on top of the vertical bars). The center dot = rMTT values associated with the maximum of DWI lesion mean deformation magnitude (= peak of Gaussian curve in Fig. 2). The limits of the vertical blue and red bars represent the lower and the upper acute rMTT values (interval [p1, p2] in Fig. 2) associated with rapidly contracting (blue) vs. expanding (red) DWI lesion areas between the acute and subacute timepoints.

In the 10 STIR stroke patients, the rMTT values in contracting or expanding DWI lesion areas were highly positively correlated (r = 0.98, p = 0.0001) (Table 1, Fig. 5). However, a larger proportion of the DWI lesion was contracting (median: 4.16% of the acute DWI lesion volume), and a smaller proportion expanding (median: 1.81% of the acute DW lesion volume) in the STIR exploratory dataset than in the derivation dataset (median: 3.39% contracting, median: 4.38% expanding), possibly reflecting the effects of thrombolysis in the STIR patients.

## 4 Discussion

We show that a dynamic metamorphosis model (Rekik et al., 2014; Trouvé and Younes, 2005; Younes, 2010) has promise for modeling ischemic stroke lesion evolution using acute and subacute DWI, rMTT and final T2-w images in space and time. This enabled us to visualize and extract dynamic features of the ischemic lesion, such as the magnitude of contraction and expansion of the DWI lesion as a function of lesion volume and in relation to rMTT values.

In our heterogeneous, small, but representative samples, we found that (i) dynamic changes in the DWI lesion were not confined to the first few hours after stroke but continued for days or weeks, accompanied by wide-ranging perfusion values; (ii) a similar wide range of perfusion values were associated with large DWI lesion deformations from acute to subacute timepoints within individual patients, meaning that in most patients (75%) the rMTT values covered the same range in contracting and expanding DWI regions; in about 25% of patients in both datasets, the perfusion values were higher in contracting than expanding DWI regions; (iii) there was large variation between patients in the perfusion values in DWI lesion areas that undergo the largest deformations, even where there was greater DWI contraction after thrombolysis; and (iv) there was large between-patient variation in the amount of DWI lesion change in acute to final phases after stroke, although we found more DWI lesion contraction in patients in STIR who received thrombolytic treatment than in the observational study where no patients received thrombolysis.

Our findings suggest that PWI values are more heterogeneous than has been suggested using average values obtained from DWI and PWI data obtained from regions of interest at individual timepoints (Dani et al., 2012). The variation is consistent with the wide variation in perfusion levels found in the literature (Kane et al., 2007; Dani et al., 2012), and with the recent concept of perfusion strata (or confidence intervals) as a biologically plausible representation of infarct risk maps (Nagakane et al., 2012). The absence of a clear difference between perfusion values in expanding versus contracting DWI lesion areas in 75% of our small but representative group of 30 patients points to a need to identify other factors that influence DWI lesion progression or reversal. The influence of lesion swelling, perfusion heterogeneity at capillary level (Ostergaard et al., 2013), collaterals, completeness of arterial occlusion at the primary occlusion site (Rekik et al., 2012; Phan et al., 2009) and perfusion levels assessed with other perfusion parameters, should be examined in future studies.

Since our sample lacked statistical power due to its small size, the method should be applied in larger datasets of stroke patients with varied lesions, timepoints, treatments and outcomes and with other perfusion parameters. Although we did not explicitly model the effect of arterial patency, the perfusion being delivered to the tissues was captured in the voxel-level rMTT values. These observations of stroke lesion ‘natural history’ in the 20-patient derivation dataset and ‘thrombolysis-enhanced’ in the 10 patient exploratory dataset, highlight the complexity and the variability of ischemic stroke lesion dynamics (Scalzo et al., 2013). Further refinement of the 4D model and inclusion of other factors such as recanalization and lesion swelling would further advance our understanding of these dynamics, explain inconsistencies between past studies (Dani et al., 2012), and provide a more nuanced understanding of how perfusion values influence DWI lesion progression to different fates.

## 5 Conclusion

In this work, we used the metamorphosis model that tracks both intensity and shape changes in evolving images to examine the influence of local voxel-wise perfusion values on ischemic lesion core dynamics (i.e. contraction and expansion) visible on diffusion weighted imaging. The wide range of perfusion values and lack of difference between contracting and expanding areas emphasizes the very dynamic nature of stroke and explains difficulty in using threshold values to discriminate tissues states. Indeed, the noted observations add up to the growing evidence of the wide spectrum of heterogeneous perfusion values, and question the true extent of their contribution into determining final infarcted tissue boundary. As noted in (Rekik et al., 2012), we believe that applying highly advanced, accurate and robust medical image analysis methods will help neurologists and stroke researchers converge to a unified vision of stroke dynamics and what truly drives tissue death. More clinical factors remain broadly unknown and others can be included into honing these models in future studies (e.g., swelling or spontaneous reperfusion). Patient-specific dynamic modeling could be of potential use in future larger studies to determine what factors influence stroke lesion evolution and responses to treatment in individual patients. Such advances are necessary to determine in future which patients are most suited to which treatments — i.e. personalized medicine.

## Figures and Tables

**Table 1:** For contraction and expansion rMTT maxima, each column successively shows their min, max and mean ± standard deviation values across patients in two different datasets. For DWI lesion volumetric proportion of highly dynamic areas, we show the min, max and median values. ^⁎^Highly contracting areas are voxels within the DWI lesion boundary whose speed of contraction is higher than the mean of the speed of contraction within the DWI lesion minus its standard deviation. Highly expanding areas are voxels of the DWI lesion whose speed of expansion is higher than the mean of the speed of contraction of the DWI lesion plus its standard deviation. ^†^Acute–subacute phase defines the metamorphosis of acute DWI lesion into subacute DWI lesion and subacute-final phase defines the metamorphosis of the latter into final T2-w at ≥ 1 month.

		Derivation data		STIR data
		Acute-subacute phase^†^	Subacute-final phase^†^	Acute-subacute phase
Contraction rMTT value	Min	0.1	0.85	0.91
	Max	2.17	2.12	2.72
	Mean ± std	0.74 ± 0.63	1.36 ± 0.47	1.34 ± 0.55
Expansion rMTT value	Min	0.1	0.73	0.8
	Max	3.18	2.02	2.94
	Mean ± std	0.8 ± 0.82	1.33 ± 0.41	1.32 ± 0.32
DWI lesion volumetric proportion of highly contracting areas^⁎^ (%)	Min	0.28	0.33	0
	Max	9.09	7.98	13.08
	Median	3.39	3.25	4.16
DWI lesion volumetric proportion of highly expanding areas^⁎^ (%)	Min	0.36	0.42	0
	Max	12.61	14.95	8.73
	Median	4.38	3.69	1.81
